# Changes in injecting versus smoking heroin, fentanyl, and methamphetamine among people who inject drugs in San Diego, California, 2020–2023

**DOI:** 10.1016/j.drugalcdep.2024.111318

**Published:** 2024-04-26

**Authors:** William H. Eger, Daniela Abramovitz, Angela R. Bazzi, Annick Bórquez, Carlos F. Vera, Alicia Harvey-Vera, Joseph R. Friedman, Steffanie A. Strathdee

**Affiliations:** a School of Social Work, San Diego State University, San Diego, CA, USA; b School of Medicine, University of California San Diego, La Jolla, CA, USA; c Herbert Wertheim School of Public Health, University of California San Diego, La Jolla, CA, USA; d Boston University School of Public Health, Boston, MA, USA; e Center for Social Medicine and Humanities, University of California, Los Angeles, Los Angeles, USA

**Keywords:** Injection drug use, Epidemiology, Fentanyl, Smoking, Methamphetamine, Heroin

## Abstract

**Background:**

Amidst an increasingly toxic drug supply in North America, people who inject drugs may be transitioning to smoking them. We aimed to assess changes in injecting and smoking opioids and methamphetamine among a cohort of people who inject drugs from San Diego, California.

**Methods:**

Over five six-month periods spanning October 2020–April 2023, we assessed prevalence of injecting and smoking opioids or methamphetamine and whether participants used these drugs more frequently by smoking than injecting. Multivariable Poisson regression via generalized estimating equations was used to examine time trends.

**Results:**

Of 362 participants, median age was 40 years; a minority were female (29%), Hispanic/Latinx/Mexican (45%), and housed (33%). Among this cohort, of whom 100% injected (and 84% injected and smoked) in period one (October 2020-April 2021), by period five (November 2022-April 2023), 34% only smoked, 59% injected and smoked, and 7% only injected. By period five, the adjusted relative risk (aRR) of injecting opioids was 0.41 (95% Confidence Interval [CI]: 0.33, 0.51) and the aRR for injecting methamphetamine was 0.50 (95% CI: 0.39, 0.63) compared to period one. Risks for smoking fentanyl rose significantly during period three (aRR=1.44, 95% CI: 1.06, 1.94), four (aRR=1.65, 95% CI: 1.24, 2.20) and five (aRR=1.90, 95% CI: 1.43, 2.53) compared to period one. Risks for smoking heroin and methamphetamine more frequently than injecting these drugs increased across all periods.

**Conclusions:**

Opioid and methamphetamine injection declined precipitously, with notable increases in smoking these drugs. Research is needed to understand the health consequences of these trends.

## 1. Introduction

Since 2015, rising rates of polysubstance use between illicitly manufactured fentanyls and stimulants, such as methamphetamine, have been recognized as a ‘fourth wave’ of the overdose crisis (Friedman and Shover, 2023). Widespread contamination and replacement of heroin with fentanyl has caused drug-related overdose deaths to surge between 2013 and 2022, resulting in nearly 108,000 deaths in 2022 (Friedman and Shover, 2023; National Institute on Drug Abuse, 2023; Spencer et al., 2024), and infectious disease transmission attributed to injection drug use has risen nationally (Centers for Disease Control and Prevention, 2020, 2018; Wong et al., 2021). Polysubstance use, meanwhile, has compounded health challenges, with California’s rate of overdose deaths doubling between 2018 (12.8 per 100,000) and 2021 (26.6 per 100,000) (Friedman and Shover, 2023; Spencer et al., 2024; Gladden et al., 2019; Mattson et al., 2021; Centers for Disease Control and Prevention, 2022).

Emerging evidence, particularly along the West Coast of North America, suggests that many people who previously injected heroin have transitioned to smoking fentanyl (Kral et al., 2021; Valasek et al., 2023; Kamal et al., 2023; Parent et al., 2021). For example, among people using opioids in San Francisco, California, there was a significant decline in injecting heroin with a simultaneous increase in daily fentanyl smoking between 2018 and 2020 (Kral et al., 2021). Research from British Columbia from 2019 and 2021 also documented rising preferences for smoking opioids among people accessing harm reduction services (Kamal et al., 2023; Parent et al., 2021). In addition to individual preferences, characteristics of the drug supply can play a critical role in one’s decision to inject or smoke, indicating that the rapidly evolving and volatile drug supply across North America may be partially responsible for these emerging trends (Valasek et al., 2023; Harris et al., 2015; Syvertsen et al., 2016) and that they might not be isolated to the West Coast.

Transitioning from injecting to smoking is hypothesized to yield several positive health and social outcomes, including reduced incidence of blood-borne infectious diseases and skin infections, substance use disorder severity, and stigma (Kral et al., 2021; Fischer et al., 2006; Harris, 2020; Leonard et al., 2008; Megerian et al., 2023). However, the extent to which transitioning from injecting to smoking drugs is occurring in the context of polysubstance use involving different types of unregulated opioids and stimulants is unknown because previous studies have not differentiated between heroin and fentanyl or assessed potential changes in the use of common stimulants such as methamphetamine. Understanding trends in the consumption of these specific drugs is vitally important in contexts where fentanyl has nearly replaced heroin and in regions where overdose deaths increasingly involve both opioids and stimulants. As recent data suggests that smoking unregulated drugs is increasingly involved in U.S. overdose deaths, there is an urgent need to understand the extent of these transitions and potential opportunities for intervention, including the delivery of harm reduction services to people who smoke (Harris, 2020; Tanz et al., 2024). To inform improved research and public health programmatic efforts, we assessed trends in injecting and smoking heroin, fentanyl, and methamphetamine in a cohort of people who injected drugs at enrollment in San Diego, California, where the local drug supply has rapidly evolved in the context of ongoing cross-border drug trafficking and regional patterns of polysubstance use between opioids and methamphetamine (Bailey et al., 2022; Brouwer et al., 2006).

## 2. Materials and methods

### 2.1. Participants and eligibility

Participants were from the *La Frontera* cohort study, an ongoing, prospective investigation of HIV, Hepatitis C and drug overdose outcomes in the context of binational drug markets and cross-border mobility between San Diego, United States, and Tijuana, Mexico (Strathdee et al., 2021). This region is critical to understanding the evolving U.S. drug supply and its ramifications given a significant portion of the country’s opioid supply originates from Mexico and border cities serve as transit hubs for the unregulated drug supply into the U.S (Drug Enforcement Administration, 2020; Friedman et al., 2022). Eligible participants were aged ≥18 years, reported past-month injection drug use (as evidenced through injection stigmata) and were living in San Diego County (hereafter: San Diego) or Tijuana at the time of screening. Half of the San Diego sample was intentionally recruited to reflect those who had crossed the border to use drugs in Tijuana within the past two years. Due to regional differences in fentanyl penetration and access to harm reduction services that may impact trends in injecting and smoking heroin, fentanyl, and methamphetamine (Friedman et al., 2022; Chen et al., 2018), this analysis was limited to participants who resided in San Diego who provided data between October 28, 2020, (first enrollment date) and April 27, 2023 (corresponding to five follow-up periods).

### 2.2. Data collection

Trained bilingual interviewers collected data via a mobile outreach van that frequented locations with a high prevalence of drug use. Baseline recruitment occurred in two waves, October 28, 2020, to October 25, 2021 (Cohort 1; n=254) and February 7, 2022, to June 9, 2022 (Cohort 2; n=108). Following eligibility screening, participants provided written informed consent and completed interviewer-administered structured surveys at baseline, six-, 12-, 18-, and 24-months, lasting about 1 hour (see Supplemental Table 1). Participants received $20 USD compensation for each assessment. All study activities were approved by Institutional Review Boards at the University of California San Diego and Universidad Xochicalco in Tijuana.

### 2.3. Survey measures

At each semi-annual visit, structured interviews gathered detailed data on sociodemographic characteristics, substance use behaviors and experiences of non-fatal overdose, as described below.

*Outcomes of interest* included 1) prevalence of injecting and smoking heroin, fentanyl, or methamphetamine in the past six months; and 2) whether participants used these drugs more frequently by smoking than injecting. Each survey asked: 1) “During the last six months, on average, how often did you inject [drug]?” and 2) “During the last six months, on average, how often did you smoke, inhale, snort, or vape [drug]?” Responses included: never; one time per month or less; 2–3 days per month; one time per week; 2–3 days per week; 4–6 days per week; one time per day; 2–3 times per day; and ≥4 times per day. Participants could respond affirmatively to using any of the three drugs alone or in combination or through multiple routes; however, we did not examine polydrug or poly-route use explicitly.

We first examined whether participants who used at least one of the three drugs (heroin, fentanyl, or methamphetamine) only injected, smoked or injected, and only smoked any of the three drugs in the past six months by study period to determine the prevalence of injecting and smoking any drug over time. We then created eight binary variables corresponding to 1) whether a participant injected or smoked heroin, fentanyl, opioids (heroin and/or fentanyl use), or methamphetamine in the past six months for each period. Heroin and fentanyl use were combined to assess overall patterns in opioid use due to the replacement of heroin with fentanyl in the U.S.-Mexico border region during our study period, which may have led to misclassification (Friedman et al., 2022). For our second outcome, we created binary variables that indicated, among participants who used heroin, fentanyl, and/or methamphetamine in the previous six months, 2) whether participants used the drug more frequently via smoking than injecting.

*Our primary predictor of interest* was time period. We divided the data chronologically, regardless of study visit, into five mutually exclusive and consecutive six-month periods: 1) October 28, 2020, to April 2021, 2) May 2021 to October 2021, 3) November 2021 to April 2022, 4) May 2022 to October 2022, and 5) November 2022 to April 27, 2023.

*Additional variables* included age in years at baseline, sex assigned at birth (female or male), ethnicity (non-Hispanic/Latino or Hispanic/Latino/Mexican), country of birth (U.S. or other), years of education completed, monthly income (≥500 USD or <500 USD), past six-month housing status (housed or unhoused), incarceration status (no or yes), receptive needle sharing (no or yes), non-fatal overdose experience (no or yes) and use of methadone, buprenorphine, or “other drug treatment” (including rehabilitation centers or other programs that help reduce substance use) (no or yes). We also described years of injection drug use, current smoking of cigarettes (no or yes), and past six-month marijuana use (no or yes), though these variables were not included in multivariable models.

### 2.4. Statistical analyses

We first described the sample according to baseline sociodemographic characteristics, substance use behaviors (e.g., past six-month injecting and smoking of heroin, fentanyl, and methamphetamine) and non-fatal overdose experiences by calculating frequencies and percentages for categorical variables and medians and interquartile ranges for continuous variables. Frequencies and percentages were used to describe the distribution of use and whether participants used more frequently via smoking than injecting for each period. We also examined, among participants who used at least one of the three drugs, whether participants only injected, only smoked and injected and smoked (any of the three drugs) in the past six months by study period to determine the prevalence of injecting and smoking any drug over time.

Next, to assess differential attrition in baseline characteristics between participants who completed at least one follow-up visit and those who did not, we used Chi-Square, Fisher’s exact and Mann-Whitney tests (for binary and continuous variables, respectively). We also conducted a similar supplemental analysis with baseline characteristics to compare each recruitment wave, which revealed that cohort one differed slightly from cohort two (p<0.05; data not shown). Due to these differences by cohort, we controlled for “recruitment wave” in multivariable models.

We then used multivariable Poisson regressions via generalized estimating equations with robust standard errors and an unstructured covariance matrix to assess potential changes in use of each drug over time (Chen et al., 2018; Zou, 2004). Time period was the primary predictor, with later periods being compared to period one concerning the risk of using each drug by 1) injecting or smoking and by 2) smoking more often than injecting. All multivariable models were adjusted for recruitment wave, age, sex assigned at birth and past six-month use of methadone, buprenorphine, or “other drug treatment”, which were selected *a priori* based on the literature (Kral et al., 2021). Interpretations were based on the magnitude of the adjusted relative risk (aRR) and corresponding 95% confidence intervals (Sterne, 2001; Amrhein et al., 2019). All analyses were conducted using SAS version 9.4; graphs were made in R version 4.3.2.

## 3. Results

### 3.1. Sample characteristics

The analytical sample consisted of 362 unique participants who completed ≥1 study visit during the study period, comprising 876 surveys and a median of two visits per participant (interquartile range [IQR]=2.0, 4.0). Most (77%) had more than one study visit before the cut-off date for this analysis. Approximately 6% (n=21) died before April 31, 2023, and did not contribute follow-up data. Those who did not have more than one study visit were younger (p=0.03), but no other significant differences emerged between the groups.

At baseline, median age was 40 years (IQR=33.0, 52.0; Table 1). A minority were born in another country (5%), assigned female sex at birth (29%) and were Hispanic/Latino/Mexican (45%). Two-thirds (67%) reported being unhoused and 15% were incarcerated in the past six months. At baseline, most injected heroin (72%) and methamphetamine (70%) and also smoked, snorted, inhaled or vaped methamphetamine (81%) in the past six months. The past six-month prevalence of receptive syringe sharing (34%) and non-fatal overdose (20%) was high, while only 13% were enrolled in methadone, buprenorphine, or “other drug treatment”.

### 3.2. Prevalence of injecting only, smoking only, or injecting and smoking

**Fig. 1.**
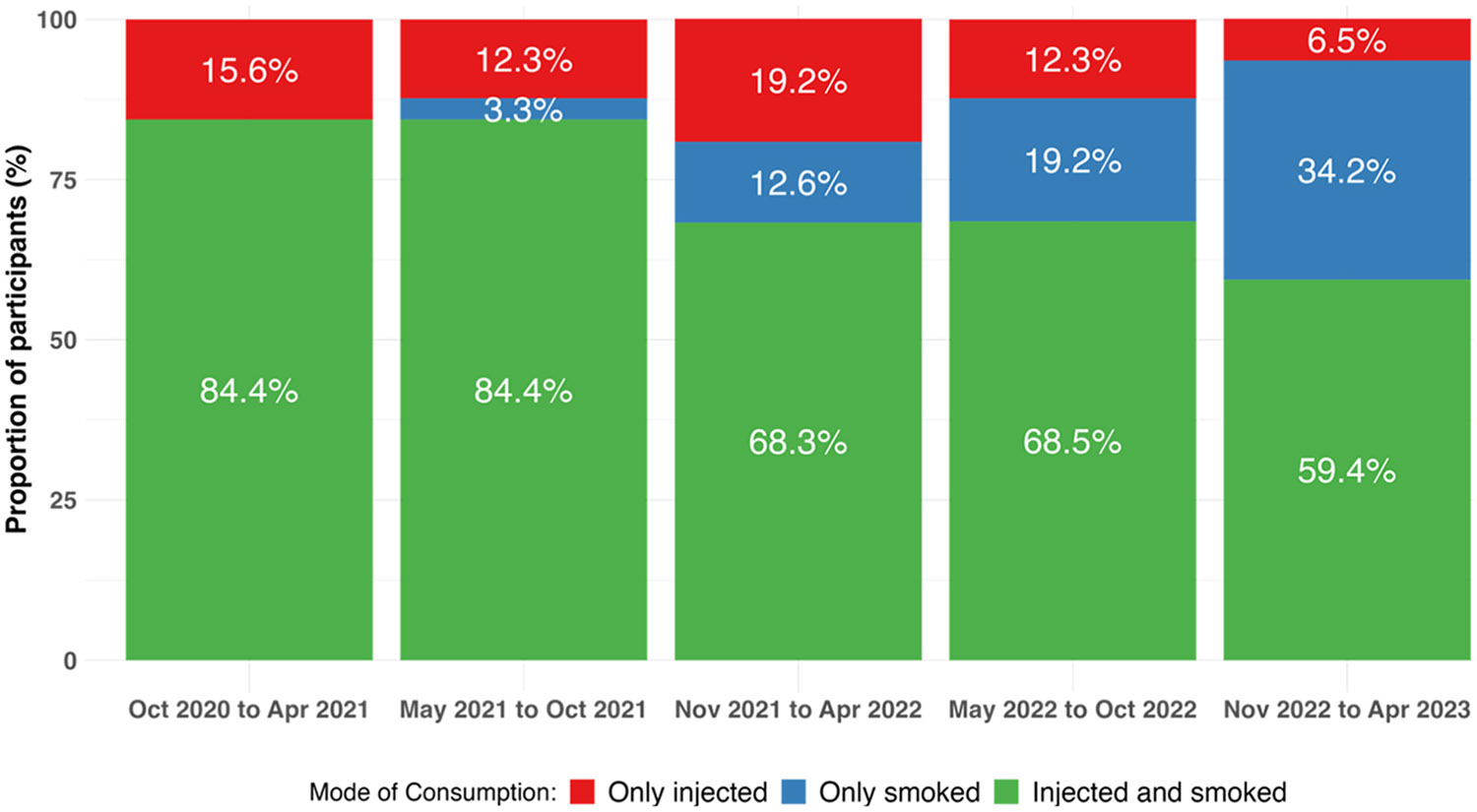
Prevalence of injecting only, smoking only, and injecting and smoking (heroin, fentanyl, or methamphetamine) in the past six months among study participants residing in San Diego County, California, who used heroin, fentanyl, or methamphetamine in the past six months between October 28, 2020, and April 27, 2023. Abbreviations: Oct = October; Nov = November; Apr = April.

As represented in Fig. 1 (and Supplemental Table 2), the prevalence of only injecting decreased over time, dropping from 15.6% in period one to 6.5% in period five. Additionally, the prevalence of injecting and smoking decreased over time, falling from 84.4% of the sample in period one to 59.4% in period five. Conversely, the fraction of participants only smoking increased over time, with none reporting this behavior in period one (by definition, as only people who injected drugs were included in the study) and 34.2% reporting it in period five.

### 3.3. Prevalence of injecting and smoking heroin, fentanyl, opioids, or methamphetamine among the total sample

**Fig. 2.**
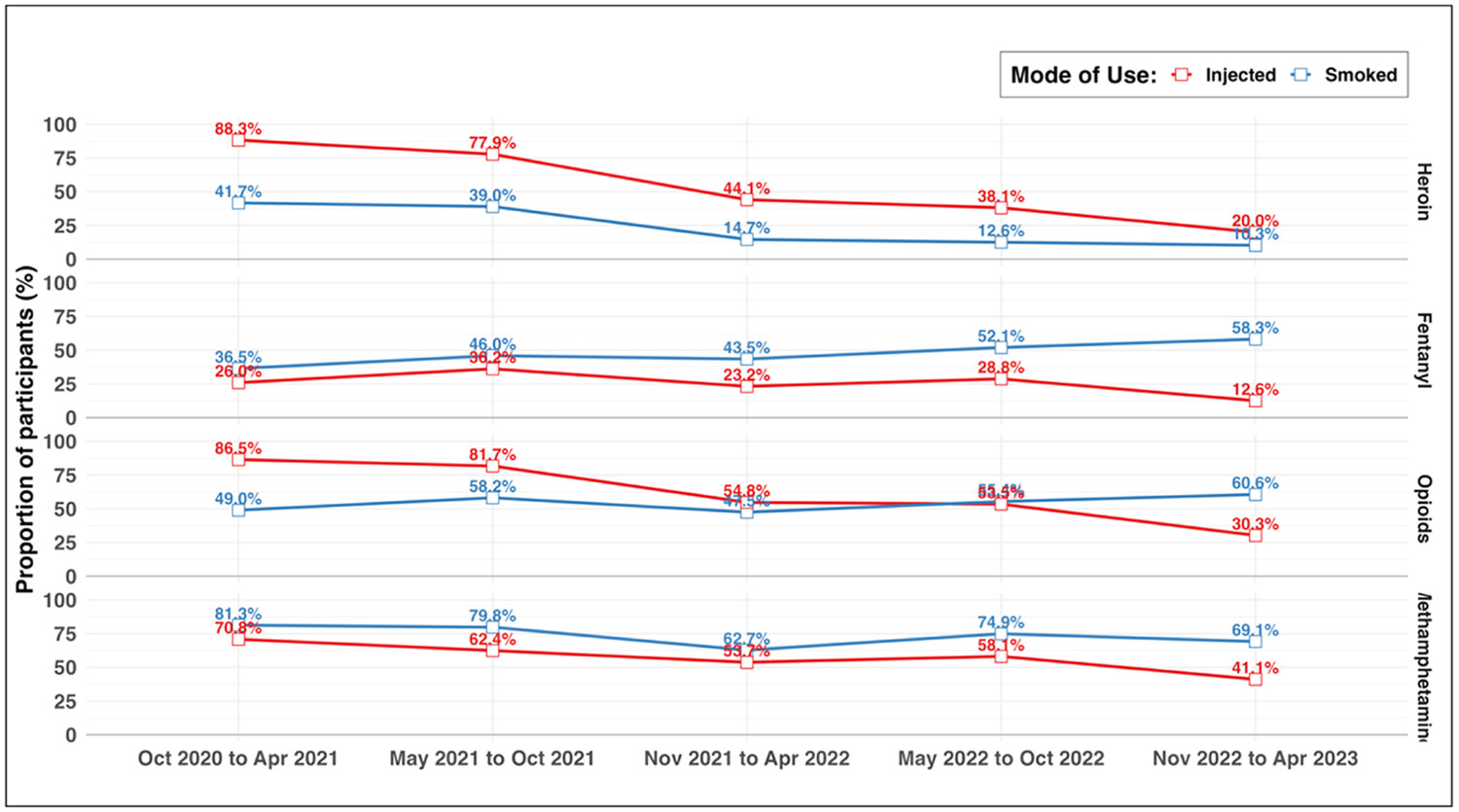
Prevalence of injecting or smoking heroin, fentanyl, opioids, or methamphetamine among study participants residing in San Diego County, California, between October 28, 2020, and April 27, 2023. Abbreviations: Oct = October; Nov = November; Apr = April; opioids = use of heroin or fentanyl in the past six months.

As shown in Table 2 and corresponding Fig. 2, the risk of using heroin decreased significantly in period five (vs. period one; aRR=0.28; 95% confidence interval [CI]: 0.21, 0.39). The risk of injecting heroin in the past six months also declined by 46% in period three (aRR=0.54; 95% CI: 0.44, 0.66), 54% in period four (aRR=0.46; 95% CI: 0.38, 0.57) and 76% in period five (aRR=0.24; 95% CI: 0.17, 0.34) compared to period one. Similarly, the risk of smoking heroin in the past six months declined in periods three (aRR=0.42; 95% CI: 0.27, 0.67), four (aRR=0.35; 95% CI: 0.23, 0.54), and five (aRR=0.30; 95% CI: 0.17, 0.50) relative to period one.

The risk of using fentanyl increased by 34% in period three (aRR=1.34; 95% CI: 1.04, 1.73), 49% in period four (aRR=1.49; 95% CI: 1.17, 1.90), and 52% in period five (aRR= 1.52; 95% CI: 1.19, 1.96). The prevalence of injecting fentanyl was stable across periods one through four but was 57% lower in period five (vs. period one; aRR=0.43; 95% CI: 0.25, 0.74). In contrast, the risk of smoking fentanyl in the past six months increased by 44% in period three (aRR=1.44; 95% CI: 1.06, 1.94), 65% in period four (aRR=1.65; 95% CI: 1.24, 2.20), and 90% in period five (aRR=1.90; 95% CI: 1.43, 2.53) compared to period one.

The risk of using opioids was stable from periods one to four but decreased by 13% in period five (vs. period one; aRR=0.87; 95% CI: 0.76, 0.98). The risk of injecting opioids declined by 31% in period three (aRR=0.69; 95% CI: 61, 0.78), 33% in period four (aRR=0.67; 95% CI: 0.60, 0.76), and 59% in period five (aRR=0.41; 95% CI: 0.33, 0.51). The risk of smoking opioids, meanwhile, increased by 25% in period three (aRR=1.25; 95% CI: 1.00, 1.56), 39% in period four (aRR=1.39; 95% CI: 1.13, 1.72), and 69% in period five (aRR=1.69; 95% CI: 1.37, 2.07) relative to period one.

The risk of methamphetamine use also decreased over time, with a 21% decline in risk in period five (vs. period one; aRR=0.79; 95% CI: 0.70, 0.89). The risk of injecting and smoking methamphetamine also decreased, with a more marked decline in the risk of injecting than smoking. For instance, in period five, the risk of injecting methamphetamine decreased by 50% (vs. period one; aRR=0.50; 95% CI: 0.39, 0.63) while the risk of smoking methamphetamine decreased by 17% (vs. period one; aRR=0.83; 95% CI: 0.71, 0.96).

### 3.4. Smoking heroin, fentanyl opioids, or methamphetamine more often than injecting

**Fig. 3.**
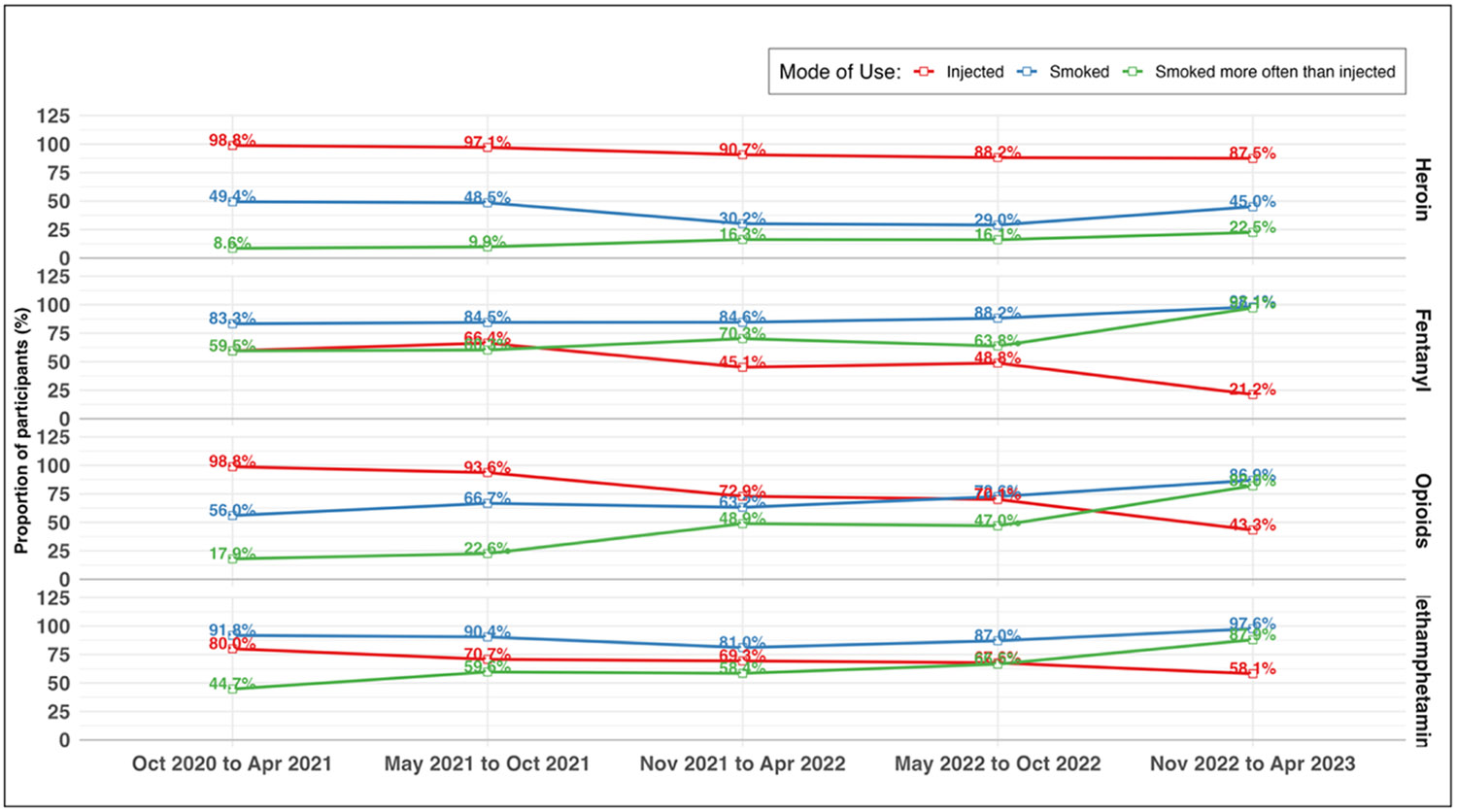
Prevalence of smoking heroin, fentanyl, opioids, or methamphetamine more often than injecting among study participants residing in San Diego County, California, who used heroin, fentanyl, or methamphetamine in the past six months, between October 28, 2020, and April 27, 2023. Abbreviations: Oct = October; Nov = November; Apr = April; opioids = use of heroin or fentanyl in the past six months.

Among participants who used heroin (Table 3 and Fig. 3), the risk of injecting it declined by 14% between periods one and five (aRR=0.86; 95% CI 0.77, 0.97). The prevalence of smoking heroin remained stable over time, with no significant changes from period one over the study period. However, relative to period one, the risk of smoking heroin more often than injecting it increased by 169% in period three (aRR=2.69; 95% CI: 1.11, 6.49), 158% in period four (aRR=2.58; 95% CI: 1.08, 6.16) and 249% in period five (aRR=3.49; 95% CI: 1.39, 8.74).

Among participants who used fentanyl, risk of injecting it decreased by 71% from period one to period five (aRR=0.29, 95% CI 0.18, 0.46). Meanwhile, prevalence of smoking fentanyl remained high and stable between periods one and four, with risk of smoking fentanyl increasing by 26% in period five (vs. period one; aRR=1.26; 95% CI: 1.09, 1.46). In period five, risk of smoking fentanyl more often than injecting it increased by 80% (vs. period one; aRR=1.80, 95% CI 1.39, 2.33).

Among participants who used any opioids, risk of injecting them decreased by 59% from period one to period five (aRR=0.41; 95% CI: 0.33, 0.51). Risk of smoking opioids, meanwhile, increased substantially from period one, increasing in risk by 25% in period three (aRR=1.25; 95% CI: 1.00, 1.56), 39% in period four (aRR=1.39; 95% CI: 1.13, 1.72), and 69% in period five (aRR=1.69; 95% CI: 1.37, 2.07). Risk of smoking more often than injecting opioids also increased in periods three (aRR=3.02; 95% CI: 1.86, 4.92), four (aRR=2.83, 95% CI: 1.74, 5.49) and five (aRR=4.99; 95% CI: 3.13, 7.96) relative to period one.

Among participants who used methamphetamine, risk of injecting it declined by 23% in period three (aRR=0.77; 95% CI: 0.64, 0.91), 24% in period four (aRR=0.76; 95% CI: 0.64, 0.89) and 48% in period five (aRR=0.52; 95% CI: 0.52, 0.77) compared to period one. Risk of smoking methamphetamine declined by 13% in period three (vs. period one; aRR=0.87; 95% CI: 0.78, 0.97) but returned to period one levels in periods four and five. Risk of smoking methamphetamine more often than injecting it increased by 34% in period two (aRR=1.34; 95% CI: 1.03, 1.74), 43% in period three (aRR=1.43; 95% CI: 1.08, 1.89), 61% in period four (aRR=1.61; 95% 1.24, 2.08), and 118% in period five (aRR=2.18; 95% CI: 1.70, 2.79) relative to period one.

## 4. Discussion

This longitudinal assessment of drug consumption patterns among a cohort of people who injected drugs at baseline identified significant declines in the injection of opioids and methamphetamine from October 2020 to April 2023. We also observed marked increases in the prevalence of smoking these drugs and the likelihood of smoking these drugs more often than injecting them. These findings provide additional nuance on changing drug consumption patterns on the West Coast of North America and have potential implications for future research and public health programming for people who use drugs. These revelations may be especially important in the context of rising overdose deaths driven by smoking drug use and polysubstance use with opioids and stimulants nationally (Friedman and Shover, 2023; Tanz et al., 2024; Ciccarone, 2021).

Our study expands upon national and regional literature noting the diminishing prevalence of heroin use due to fentanyl’s infiltration into the drug supply and highlights a pronounced reduction in heroin use over time (Friedman et al., 2022; Ciccarone, 2017; Fleiz et al., 2020). We found a marked decline in both heroin injection and smoking in the overall sample, coupled with substantial increases in the risk of smoking heroin more often than injecting it over time among those recently using heroin.

In contrast, consistent with recently reported findings from San Francisco (Kral et al., 2021), fentanyl use patterns were more complex. Fentanyl injection, for instance, remained somewhat stable before decreasing significantly among those recently reporting its use. Meanwhile, fentanyl smoking increased overall, with a growing inclination towards smoking it more often than injecting it among those reporting its use. The observed increases in opioid smoking behaviors were likely primarily influenced by the rapid replacement of heroin with fentanyl in the U.S.-Mexico border region during our study period, where fentanyl positivity rates in collected syringes increased by 21.7% (Friedman et al., 2022). The emergence of fentanyl may have driven some individuals to transition from injecting to mitigate health challenges that have been exacerbated by fentanyl, such as poor vein health, skin infections, and overdose (Kral et al., 2021; Megerian et al., 2023). Surely the heightened potency of fentanyl may facilitate a strong effect when smoking. However, a lack of consistently available drug-checking services in San Diego for most of our study period may have led some participants to misclassify heroin (or fentanyl) use when, in reality, they were using something else (Bailey et al., 2023). The latter might further explain the shift from injecting to smoking in the context of heroin use. Altogether, our results imply an ongoing trend where injection of opioids is being supplanted by smoking.

This study examined previously unexplored patterns of methamphetamine consumption behaviors that extend our understanding of evolving polysubstance use trends. Methamphetamine injection declined by 50% over the study period and prevalence of smoking methamphetamine also declined overall (though less so than injecting). This could reflect behaviors amongst a fixed cohort rather than a population-level trend. Notably, smoking methamphetamine remained a common behavior in our sample and the overall pattern of transitioning from injecting to smoking was especially prominent for participants who used methamphetamine, where we saw a 100% increase in smoking methamphetamine more often than injecting it by the end of the study period. This trend may reflect the growing prevalence of polysubstance use involving methamphetamine and fentanyl (Han et al., 2021; Havens et al., 2021; Ahmed et al., 2022), whereby methamphetamine may be used in combination to mitigate the heightened sedative effects of fentanyl, better cope with a more rapid onset of withdrawal symptoms and improve euphoric effects (Ondocsin et al., 2023; Lopez et al., 2021; Rudolph et al., 2024). Also, the overall increase in smoking could potentially be due to the rising potency of illicitly manufactured fentanyls and methamphetamines that can create a strong effect that was not previously possible with heroin alone (Ondocsin et al., 2023). Additional research explicitly examining preferences and behaviors involved in co-use of opioids and methamphetamine is needed; however, caution is advised for research on preferences, as drug consumption is largely determined by drug market dynamics rather than individual choice (Ali et al., 2021; Collins et al., 2024).

The decreases in injecting and increases in smoking that we observed across these three unregulated drugs, which are commonly used together, may reflect changing community norms and preferences as well as prevention efforts focused on blood-borne infectious diseases (e.g., HIV, Hepatitis C, skin and soft tissue infections) and vein health (Kral et al., 2021; Valasek et al., 2023; Leonard et al., 2008; Megerian et al., 2023). Research is urgently needed to identify and quantify the positive and negative health impacts of smoking compared to injecting these drugs, as long-term risks may offset short-term benefits. For instance, while previous work on injection cessation and route transition interventions (such as those encouraging individuals to transition from injecting to smoking) highlights reduced syringe sharing and injection frequency (Bridge, 2010; Fitzpatrick et al., 2022; Dunleavy et al., 2021; Stöver and Schäffer, 2014), the precise public health impacts of transitioning from injecting to smoking on blood-borne infectious disease transmission and overdose rates remain largely unknown, and possible harms to cardiovascular and lung health remain understudied (Koslik et al., 2020). Without such evidence, widespread beliefs that smoking (versus injecting) is “safer” could produce a sense of complacency and fewer harm-reducing behaviors (e.g., not carrying naloxone, increasing use while alone). Furthermore, the frequency of injections versus inhalation of fentanyl and methamphetamine—and related impacts on the length of time to onset of withdrawal symptoms among dependent individuals—deserves careful study. Indeed, it is possible that transitions to smoking lead to more frequent use and, hence, an increased risk of overdose. Ultimately, intervention and implementation research should aim to investigate strategies that promote equitable access to safer smoking supplies and other route transition interventions paired with harm reduction education, which could help connect people who smoke drugs to necessary services (Tanz et al., 2024; Singh SB-G and Kingston, 2022).

In interpreting the results of this study, we acknowledge several limitations. First, reliance on self-reported behaviors introduces potential for misclassification, such as fentanyl being erroneously classified as heroin. Nevertheless, even in the presence of potential misclassification, the overall trends in injection remained unaffected, lending support to our interpretations. Second, trends for opioids (heroin and/or fentanyl) should be interpreted with caution since declines in opioid use are likely driven by decreased heroin use, while the increase in opioid smoking (and decrease in opioid injecting) is likely driven by changing fentanyl consumption behaviors. These differences indicate the importance of examining heroin and fentanyl use separately. Also, while polysubstance use dynamics were not explicitly assessed, there was likely a high prevalence of these behaviors in our sample, warranting additional investigation. Third, while efforts were made to minimize retention bias, the study experienced some loss to follow-up. Although we found minimal differences in baseline sociodemographics and substance use behaviors between those who completed at least one follow-up visit and those who did not, caution should be exercised in extrapolating these results to broader populations. Similarly, our results may not be generalizable to other contexts with other drug market dynamics. However, trends observed here may offer early insights into shifts that may subsequently occur in other regions, particularly along the West Coast of North America, which is a crucial transit point for unregulated drugs (Drug Enforcement Administration, 2020; Ciccarone, 2017).

### 4.1. Conclusions

This longitudinal assessment of people who inject drugs from San Diego, California, revealed significant declines in the injection of heroin, fentanyl, and methamphetamine between October 2020 and April 2023. Notably, a substantial proportion of participants transitioned from injecting and smoking to only smoking by the end of the study period, regardless of drug classification. Also, the risk of smoking opioids more often than injecting increased over time, suggesting shifts in preference towards smoking among people using opioids. We also documented a marked increase in smoking methamphetamine more often than injecting it by the end of the study period. Taken together, these findings extend previous research identifying decreased injection and increased smoking of opioids by adding additional nuance concerning specific polysubstance use patterns. Further in-depth qualitative and quantitative research is needed to understand the determinants of these trends and their implications for the health of substance-using populations. Efforts are needed to scale up harm reduction services, including the provision of safer smoking supplies, to address these current transitions.

## Supplementary Material

Supplementary Material

## Figures and Tables

**Table 1:** Baseline characteristics of study participants residing in San Diego County, California.

Variable	Total N (%)^a^
	N=362
**Median age (IQR)**	40.0 (33.0, 52.0)
**Sex assigned at birth**	
**Female**	103 (28.5%)
**Male**	259 (71.5%)
**Ethnicity**	
**Non-Hispanic/Latino**	142 (60.8%)
**Hispanic/Latino/Mexican**	220 (39.2%)
**Country of birth**	
**Other**	18 (5.0%)
**United States**	344 (95.0%)
**Median years of education completed (IQR)**	12.0 (10.0, 13.0)
**Monthly income**	
**≥500 USD**	218 (60.2%)
**<500 USD**	144 (39.8%)
**Housing status** *	
**Housed**	119 (32.9%)
**Unhoused**	243 (67.1%)
**Incarcerated** * $\overset{Y}{1}$	(n= 359)
**No**	306 (85.2%)
**Yes**	53 (14.8%)
**Currently smokes cigarettes**	
**No**	138 (38.1%)
**Yes**	224 (61.9%)
**Smoked or vaped marijuana** *	
**No**	136 (37.6%)
**Yes**	226 (62.4%)
**Smoked/snorted/inhaled/vaped heroin** *	
**No**	243 (67.1%)
**Yes**	119 (32.9%)
**Smoked/snorted/inhaled/vaped fentanyl** *	
**No**	217 (59.9%)
**Yes**	145 (40.1%)
**Smoked/snorted/inhaled/vaped methamphetamine** *	
**No**	70 (19.3%)
**Yes**	292 (80.7%)
**Injected heroin** *	
**No**	101 (27.9%)
**Yes**	261 (72.1%)
**Injected methamphetamine** *	
**No**	107 (29.6%)
**Yes**	255 (70.4%)
**Injected fentanyl** *	
**No**	236 (65.2%)
**Yes**	126 (34.8%)
**Median years of injection drug use (IQR)**	16.0 (8.0, 28.0)
**Receptive needle sharing** *	
**No**	239 (66.0%)
**Yes**	123 (34.0%)
**Experienced overdose** * $\overset{Y}{2}$	
**No**	288 (80.0%)
**Yes**	72 (20.0%)
**Use of methadone, buprenorphine, or “other drug Treatment”** *	
**No**	316 (87.3%)
**Yes**	46 (12.7%)

Missing values: Y_1_: n=3; Y_2_: n=2

Abbreviations: IQR = interquartile range.

a Total number (and percent) of observations at baseline (October 2020-April 2021).

* Indicates a behavior assessed in the past six months.

**Table 2:** Prevalence of injecting and smoking heroin, fentanyl, or methamphetamine among study participants residing in San Diego County, California, between October 28, 2020, and April 27, 2023.

Drug	Time Period	Obs^a^	Mode of Use N (%)	aRR^b^ (95% CI)
*Heroin*		N=876	*Injected or* *smoked* *	
	Oct 2020 to Apr 2021	97	81 (84.4%)	Ref
	May 2021 to Oct 2021	214	171 (80.3%)	0.97 (0.86, 1.08)
	Nov 2021 to Apr 2022	177	86 (48.6%)	0.60 (0.50, 0.73)
	May 2022 to Oct 2022	215	93 (43.3%)	0.53 (0.44, 0.65)
	Nov 2022 to Apr 2023	175	40 (22.9%)	0.28 (0.21, 0.39)
		N=876	*Injected* *	
	Oct 2020 to Apr 2021	96	80 (88.3%)	Ref
	May 2021 to Oct 2021	213	166 (77.9%)	0.95 (0.85, 1.07)
	Nov 2021 to Apr 2022	177	78 (44.1%)	0.54 (0.44, 0.66)
	May 2022 to Oct 2022	215	82 (38.1%)	0.46 (0.38, 0.57)
	Nov 2022 to Apr 2023	175	35 (20.0%)	0.24 (0.17, 0.34)
		N=876	*Smoked* *	
	Oct 2020 to Apr 2021	96	40 (41.7%)	Ref
	May 2021 to Oct 2021	213	83 (39.0%)	0.95 (0.72, 1.27)
	Nov 2021 to Apr 2022	177	26 (14.7%)	0.42 (0.27, 0.67)
	May 2022 to Oct 2022	215	27 (12.6%)	0.35 (0.23, 0.54)
	Nov 2022 to Apr 2023	175	18 (10.3%)	0.30 (0.17, 0.50)
*Fentanyl*		N=876	*Injected or* *smoked* *	
	Oct 2020 to Apr 2021	96	42 (43.8%)	Ref
	May 2021 to Oct 2021	213	116 (54.5%)	1.25 (0.98, 1.59)
	Nov 2021 to Apr 2022	177	91 (51.4%)	1.34 (1.04, 1.73)
	May 2022 to Oct 2022	215	127 (59.1%)	1.49 (1.17, 1.90)
	Nov 2022 to Apr 2023	175	104 (59.4%)	1.52 (1.19, 1.96)
		N=876	*Injected* *	
	Oct 2020 to Apr 2021	96	25 (26.0%)	Ref
	May 2021 to Oct 2021	213	77 (36.2%)	1.39 (0.96, 2.02)
	Nov 2021 to Apr 2022	177	41 (23.2%)	0.84 (0.54, 1.30)
	May 2022 to Oct 2022	215	62 (28.8%)	1.03 (0.68, 1.55)
	Nov 2022 to Apr 2023	175	22 (12.6%)	0.43 (0.25, 0.74)
		N=876	*Smoked* *	
	Oct 2020 to Apr 2021	96	35 (36.5%)	Ref
	May 2021 to Oct 2021	213	98 (46.0%)	1.27 (0.95, 1.69)
	Nov 2021 to Apr 2022	177	77 (43.5%)	1.44 (1.06, 1.94)
	May 2022 to Oct 2022	215	112 (52.1%)	1.65 (1.24, 2.20)
	Nov 2022 to Apr 2023	175	102 (58.3%)	1.90 (1.43, 2.53)
*Opioids* ^c^		N=876	*Injected or* *smoked* *	
	Oct 2020 to Apr 2021	96	84 (87.5%)	Ref
	May 2021 to Oct 2021	213	186 (87.3%)	1.00 (0.92, 1.09)
	Nov 2021 to Apr 2022	177	133 (75.1%)	0.93 (0.83, 1.05)
	May 2022 to Oct 2022	215	164 (76.3%)	0.93 (0.84, 1.04)
	Nov 2022 to Apr 2023	175	122 (69.7%)	0.87 (0.76, 0.98)
		N=876	*Injected* *	
	Oct 2020 to Apr 2021	96	83 (86.5%)	Ref
	May 2021 to Oct 2021	213	174 (81.7%)	0.95 (0.86, 1.06)
	Nov 2021 to Apr 2022	177	97 (54.8%)	0.64 (0.54, 0.76)
	May 2022 to Oct 2022	215	115 (53.5%)	0.63 (0.53, 0.73)
	Nov 2022 to Apr 2023	175	53 (30.3%)	0.35 (0.27, 0.46)
		N=876	*Smoked* *	
	Oct 2020 to Apr 2021	96	47 (49.0%)	Ref
	May 2021 to Oct 2021	213	124 (58.2%)	1.19 (0.95, 1.50)
	Nov 2021 to Apr 2022	177	84 (47.5%)	1.15 (0.90, 1.48)
	May 2022 to Oct 2022	215	119 (55.4%)	1.30 (1.03, 1.64)
	Nov 2022 to Apr 2023	175	106 (60.6%)	1.47 (1.16, 1.86)
*Methamphetamine*		N=876	*Injected or* *smoked* *	
	Oct 2020 to Apr 2021	96	85 (88.5%)	Ref
	May 2021 to Oct 2021	213	188 (88.3%)	1.00 (0.92, 1.09)
	Nov 2021 to Apr 2022	177	137 (77.4%)	0.87 (0.77, 0.97)
	May 2022 to Oct 2022	215	185 (86.1%)	0.96 (0.88, 1.06)
	Nov 2022 to Apr 2023	175	124 (70.9%)	0.79 (0.70, 0.89)
		N=876	*Injected* *	
	Oct 2020 to Apr 2021	96	68 (70.8%)	Ref
	May 2021 to Oct 2021	213	133 (62.4%)	0.88 (0.75, 1.04)
	Nov 2021 to Apr 2022	177	95 (53.7%)	0.67 (0.55, 0.82)
	May 2022 to Oct 2022	215	125 (58.1%)	0.73 (0.60, 0.88)
	Nov 2022 to Apr 2023	175	72 (41.1%)	0.50 (0.39, 0.63)
		N=876	*Smoked* *	
	Oct 2020 to Apr 2021	96	78 (81.3%)	Ref
	May 2021 to Oct 2021	213	170 (79.8%)	0.98 (0.87, 1.11)
	Nov 2021 to Apr 2022	177	111 (62.7%)	0.76 (0.65, 0.88)
	May 2022 to Oct 2022	215	161 (74.9%)	0.90 (0.79, 1.03)
	Nov 2022 to Apr 2023	175	121 (69.1%)	0.83 (0.71, 0.96)

Abbreviations: Ref = reference category; 95% CI: 95% confidence interval.

a Obs = number of total observations in each period (study participants within each period are unique); period one (October 2020-April 2021) includes only baseline observations, but the later periods include observations from multiple visits.

b Adjusted Relative Risk (aRR) for recruitment wave, age, gender, and recent (within the past six months) substance use treatment program enrollment.

c Represents use of heroin and/or fentanyl in the past six months.

* Affirmative response, past six months.

**Table 3:** Prevalence of smoking heroin, fentanyl, opioids, or methamphetamine more often than injecting among study participants residing in San Diego County, California, who used heroin, fentanyl, opioids, or methamphetamine in the past six months, between October 28, 2020, and April 27, 2023.

Drug	Time Period	Obs^a^	Mode of Use N (%)	aRR^b^ (95% CI)
*Heroin*		N=471	*Injected* *	
*Heroin*				
	Oct 2020 to Apr 2021	81	80 (98.8%)	Ref
	May 2021 to Oct 2021	171	166 (97.1%)	0.98 (0.95, 1.02)
	Nov 2021 to Apr 2022	86	78 (90.7%)	0.89 (0.82, 0.97)
	May 2022 to Oct 2022	93	82 (88.2%)	0.87(0.79, 0.95)
	Nov 2022 to Apr 2023	40	35 (87.5%)	0.86 (0.77, 0.97)
		N=471	*Smoked* *	
	Oct 2020 to Apr 2021	81	40 (49.4%)	Ref
	May 2021 to Oct 2021	171	83 (48.5%)	0.99 (0.76, 1.29)
	Nov 2021 to Apr 2022	86	26 (30.2%)	0.73 (0.48, 1.11)
	May 2022 to Oct 2022	93	27 (29.0%)	0.68 (0.45, 1.02)
	Nov 2022 to Apr 2023	40	18 (45.0%)	1.07 (0.70, 1.65)
		N=471	*Smoked more* *often* *than injected* *	
	Oct 2020 to Apr 2021	81	7 (8.6%)	Ref
	May 2021 to Oct 2021	171	17 (9.9%)	1.14 (0.49, 2.64)
	Nov 2021 to Apr 2022	86	14 (16.3%)	2.69 (1.11, 6.49)
	May 2022 to Oct 2022	93	15 (16.1%)	2.58 (1.08, 6.16)
	Nov 2022 to Apr 2023	40	9 (22.5%)	3.49 (1.39, 8.74)
*Fentanyl*		N=480	*Injected* *	
	Oct 2020 to Apr 2021	42	25 (59.5%)	Ref
	May 2021 to Oct 2021	116	77 (66.4%)	1.12 (0.85, 1.49)
	Nov 2021 to Apr 2022	91	41 (45.1%)	0.62 (0.43, 0.89)
	May 2022 to Oct 2022	127	62 (48.8%)	0.69 (0.50, 0.97)
	Nov 2022 to Apr 2023	104	22 (21.2%)	0.29 (0.18, 0.46)
		N=480	*Smoked* *	
	Oct 2020 to Apr 2021	42	35 (83.3%)	Ref
	May 2021 to Oct 2021	116	98 (84.5%)	1.02 (0.88, 1.20)
	Nov 2021 to Apr 2022	91	77 (84.6%)	1.08 (0.92, 1.26)
	May 2022 to Oct 2022	127	112 (88.2%)	1.12 (0.96, 1.31)
	Nov 2022 to Apr 2023	104	102 (98.1%)	1.26 (1.09, 1.46)
		N=480	*Smoked more* *often than* *injected* *	
	Oct 2020 to Apr 2021	42	25 (59.5%)	Ref
	May 2021 to Oct 2021	116	70 (60.3%)	1.03 (0.77, 1.37)
	Nov 2021 to Apr 2022	91	64 (70.3%)	1.29 (0.97, 1.72)
	May 2022 to Oct 2022	127	81 (63.8%)	1.16 (0.88, 1.54)
	Nov 2022 to Apr 2023	104	101 (97.1%)	1.80 (1.39, 2.33)
*Opioids* ^c^		N=689	*Injected* *	
	Oct 2020 to Apr 2021	84	83 (98.8%)	Ref
	May 2021 to Oct 2021	186	174 (93.6%)	0.95 (0.91, 1.00)
	Nov 2021 to Apr 2022	133	97 (72.9%)	0.69 (0.61, 0.78)
	May 2022 to Oct 2022	164	115 (70.1%)	0.67 (0.60, 0.76)
	Nov 2022 to Apr 2023	122	53 (43.3%)	0.41 (0.33, 0.51)
		N=689	*Smoked* *	
	Oct 2020 to Apr 2021	84	47 (56.0%)	Ref
	May 2021 to Oct 2021	186	124 (66.7%)	1.19 (0.96, 1.47)
	Nov 2021 to Apr 2022	133	84 (63.2%)	1.25 (1.00, 1.56)
	May 2022 to Oct 2022	164	119 (72.6%)	1.39 (1.13, 1.72)
	Nov 2022 to Apr 2023	122	106 (86.9%)	1.69 (1.37, 2.07)
		N=689	*Smoked more* *often than* *injected* *	
	Oct 2020 to Apr 2021	84	15 (17.9%)	Ref
	May 2021 to Oct 2021	186	42 (22.6%)	1.26 (0.75, 2.13)
	Nov 2021 to Apr 2022	133	65 (48.9%)	3.02 (1.86, 4.92)
	May 2022 to Oct 2022	164	77 (47.0%)	2.83 (1.74, 4.59)
	Nov 2022 to Apr 2023	122	100 (82.0%)	4.99 (3.13, 7.96)
*Methamphetamine*		N=719	*Injected* *	
	Oct 2020 to Apr 2021	85	68 (80.0%)	Ref
	May 2021 to Oct 2021	188	133 (70.7%)	0.89 (0.77, 1.02)
	Nov 2021 to Apr 2022	137	95 (69.3%)	0.77 (0.64 0.91)
	May 2022 to Oct 2022	185	125 (67.6%)	0.76 (0.64, 0.89)
	Nov 2022 to Apr 2023	124	72 (58.1%)	0.63 (0.52, 0.77)
		N=719	*Smoked* *	
	Oct 2020 to Apr 2021	85	78 (91.8%)	Ref
	May 2021 to Oct 2021	188	170 (90.4%)	0.98 (0.91, 1.07)
	Nov 2021 to Apr 2022	137	111 (81.0%)	0.87 (0.78, 0.97)
	May 2022 to Oct 2022	185	161 (87.0%)	0.94 (0.86, 1.02)
	Nov 2022 to Apr 2023	124	121 (97.6%)	1.05 (0.97, 1.13)
		N=719	*Smoked more* *often* *than injected* *	
	Oct 2020 to Apr 2021	85	38 (44.7%)	Ref
	May 2021 to Oct 2021	188	112 (59.6%)	1.34 (1.03, 1.74)
	Nov 2021 to Apr 2022	137	80 (58.4%)	1.43 (1.08, 1.89)
	May 2022 to Oct 2022	185	123 (66.5%)	1.61 (1.24, 2.08)
	Nov 2022 to Apr 2023	124	109 (87.9%)	2.18 (1.70, 2.79)

a Obs = number of observations within each period, which includes only those who used each specified drug (i.e., heroin, fentanyl, opioids, methamphetamine) in the past six months (study participants within each period are unique); period one (October 2020-April 2021) includes only baseline observations, but the later periods include observations from multiple visits.

b Adjusted Relative Risk (aRR) for recruitment wave, age, gender, and recent (within the past six months) substance use treatment program enrollment.

c Represents use of heroin and/or fentanyl in the past six months.

* Affirmative response, past six months.

## Data Availability

Data is available upon reasonable request to the Principal Investigator of *La Frontera*, Dr. Steffanie Strathdee (sstrathdee@health.ucsd.edu).

## References

[R1] Ahmed S, Sarfraz Z, Sarfraz A, 2022. Editorial: a changing epidemic and the rise of opioid-stimulant co-use. Front Psychiatry 13, 918197. 35873238 10.3389/fpsyt.2022.918197 PMC9296817

[R2] Ali F, Russell C, Nafeh F, Rehm J, LeBlanc S, Elton-Marshall T, 2021. Changes in substance supply and use characteristics among people who use drugs (PWUD) during the COVID-19 global pandemic: A national qualitative assessment in Canada. Int J. Drug Policy 93, 103237. 33893026 10.1016/j.drugpo.2021.103237 PMC9759688

[R3] Amrhein V, Greenland S, McShane B, 2019. Scientists rise up against statistical significance. Nature 567 (7748), 305–307. 30894741 10.1038/d41586-019-00857-9

[R4] Bailey K, Abramovitz D, Patterson TL, Harvey-Vera AY, Vera CF, Rangel MG, , 2022. Correlates of recent overdose among people who inject drugs in the San Diego/Tijuana border region. Drug Alcohol Depend. 240, 109644. 36179507 10.1016/j.drugalcdep.2022.109644 PMC9608984

[R5] Bailey K, Abramovitz D, Artamonova I, Davidson P, Stamos-Buesig T, Vera CF, , 2023. Drug checking in the fentanyl era: Utilization and interest among people who inject drugs in San Diego, California. Int J. Drug Policy 118, 104086. 37295217 10.1016/j.drugpo.2023.104086 PMC10527490

[R6] Bridge J, 2010. Route transition interventions: potential public health gains from reducing or preventing injecting. Int. J. Drug Policy 21 (2), 125–128. 20167464 10.1016/j.drugpo.2010.01.011

[R7] Brouwer KC, Case P, Ramos R, Magis-Rodriguez C, Bucardo J, Patterson TL, , 2006. Trends in production, trafficking, and consumption of methamphetamine and cocaine in Mexico. Subst. Use Misuse 41 (5), 707–727. 16603456 10.1080/10826080500411478 PMC2757051

[R8] Centers for Disease Control and Prevention. Viral Hepatitis Surveillance Report 2018 — Hepatitis C. Centers for Disease Control and Prevention; 2018.

[R9] Centers for Disease Control and Prevention. HIV in the United States and Dependent Areas. 2020.

[R10] Centers for Disease Control and Prevention. Drug Overdose Mortality by State. In: National Center for Health Statistics, editor. 2022.

[R11] Chen W, Qian L, Shi J, Franklin M, 2018. Comparing performance between log-binomial and robust Poisson regression models for estimating risk ratios under model misspecification. BMC Med. Res. Methodol 18 (1), 63. 29929477 10.1186/s12874-018-0519-5 PMC6013902

[R12] Ciccarone D, 2017. Fentanyl in the US heroin supply: A rapidly changing risk environment. Int J. Drug Policy 46, 107–111. 28735776 10.1016/j.drugpo.2017.06.010 PMC5742018

[R13] Ciccarone D, 2021. The rise of illicit fentanyls, stimulants and the fourth wave of the opioid overdose crisis. Curr. Opin. Psychiatry 34 (4), 344–350. 33965972 10.1097/YCO.0000000000000717 PMC8154745

[R14] Collins AB, Macon EC, Levin S, Wunsch C, Wightman RS, 2024. It gets you high as a kite but not unsick”: Characterizations of and responses to a changing local drug supply by people who use drugs in Rhode Island. Int. J. Drug Policy 127, 104391. 38490014 10.1016/j.drugpo.2024.104391 PMC11127783

[R15] Drug Enforcement Administration. 2020 National drug threat assessment. 2020.

[R16] Dunleavy K, Hutchinson SJ, Palmateer N, Goldberg D, Taylor A, Munro A, , 2021. The uptake of foil from needle and syringe provision services and its role in smoking or snorting heroin among people who inject drugs in Scotland. Int J. Drug Policy 98, 103369. 34340168 10.1016/j.drugpo.2021.103369

[R17] Fischer B, Manzoni P, Rehm J, 2006. Comparing injecting and non-injecting illicit opioid users in a multisite Canadian sample (OPICAN Cohort). Eur. Addict. Res 12 (4), 230–239. 16968998 10.1159/000094425

[R18] Fitzpatrick T, McMahan VM, Frank ND, Glick SN, Violette LR, Davis S, , 2022. Heroin pipe distribution to reduce high-risk drug consumption behaviors among people who use heroin: a pilot quasi-experimental study. Harm Reduct. J 19 (1), 103. 36138407 10.1186/s12954-022-00685-7 PMC9493152

[R19] Fleiz Arredondo, C., Chavez J, Pacheco A, Segovia LA,L, Villatoro JA, , 2020. Fentanyl is used in Mexico’s northern border: current challenges for drug health policies. Addiction 115 (4), 778–781. 31837278 10.1111/add.14934

[R20] Friedman J, Shover CL, 2023. Charting the fourth wave: Geographic, temporal, race/ethnicity and demographic trends in polysubstance fentanyl overdose deaths in the United States, 2010-2021. Addiction 118 (12), 2477–2485. 37705148 10.1111/add.16318

[R21] Friedman J, Bourgois P, Godvin M, Chavez A, Pacheco L, Segovia LA, , 2022. The introduction of fentanyl on the US-Mexico border: An ethnographic account triangulated with drug checking data from Tijuana. Int J. Drug Policy 104, 103678. 35421740 10.1016/j.drugpo.2022.103678 PMC9169560

[R22] Gladden RM, O’Donnell J, Mattson CL, Seth P, 2019. Changes in Opioid-Involved Overdose Deaths by Opioid Type and Presence of Benzodiazepines, Cocaine, and Methamphetamine - 25 States, July-December 2017 to January-June 2018. MMWR Morb. Mortal. Wkly Rep 68 (34), 737–744. 10.15585/mmwr.mm6834a2 PMC6715260 31465320

[R23] Han B, Compton WM, Jones CM, Einstein EB, Volkow ND, 2021. Methamphetamine Use, Methamphetamine Use Disorder, and Associated Overdose Deaths Among US Adults. JAMA Psychiatry 78 (12), 1329–1342. 34550301 10.1001/jamapsychiatry.2021.2588 PMC8459304

[R24] Harris M, 2020. An urgent impetus for action: safe inhalation interventions to reduce COVID-19 transmission and fatality risk among people who smoke crack cocaine in the United Kingdom. Int J. Drug Policy 83, 102829. 32595070 10.1016/j.drugpo.2020.102829 PMC7306748

[R25] Harris M, Forseth K, Rhodes T, 2015. It’s Russian roulette”: Adulteration, adverse effects and drug use transitions during the 2010/2011 United Kingdom heroin shortage. Int. J. Drug Policy 26 (1), 51–58. 25444768 10.1016/j.drugpo.2014.09.009

[R26] Havens JR, Knudsen HK, Strickland JC, Young AM, Babalonis S, Lofwall MR, , 2021. Recent Increase in Methamphetamine Use in a Cohort of Rural People Who Use Drugs: Further Evidence for the Emergence of Twin Epidemics. Front Psychiatry 12, 805002. 35069295 10.3389/fpsyt.2021.805002 PMC8777215

[R27] Kamal A, Ferguson M, Xavier JC, Liu L, Graham B, Lock K, , 2023. Smoking identified as preferred mode of opioid safe supply use; investigating correlates of smoking preference through a 2021 cross-sectional study in British Columbia. Subst. Abus. Treat. Prev. Policy 18 (1), 27. 10.1186/s13011-023-00515-4 PMC10186314 37194018

[R28] Koslik HJ, Joshua J, Cuevas-Mota J, Goba D, Oren E, Alcaraz JE, , 2020. Prevalence and correlates of obstructive lung disease among people who inject drugs, San Diego, California. Drug Alcohol Depend. 214, 108158. 32652379 10.1016/j.drugalcdep.2020.108158 PMC7331511

[R29] Kral AH, Lambdin BH, Browne EN, Wenger LD, Bluthenthal RN, Zibbell JE, , 2021. Transition from injecting opioids to smoking fentanyl in San Francisco, California. Drug Alcohol Depend. 227, 109003. 34482046 10.1016/j.drugalcdep.2021.109003 PMC10790652

[R30] Leonard L, DeRubeis E, Pelude L, Medd E, Birkett N, Seto J, 2008. “I inject less as I have easier access to pipes”: Injecting, and sharing of crack-smoking materials, decline as safer crack-smoking resources are distributed. Int. J. Drug Policy 19 (3), 255–264. 18502378 10.1016/j.drugpo.2007.02.008

[R31] Lopez AM, Dhatt Z, Howe M, Al-Nassir M, Billing A, Artigiani E, , 2021. Co-use of methamphetamine and opioids among people in treatment in Oregon: A qualitative examination of interrelated structural, community, and individual-level factors. Int J. Drug Policy 91, 103098. 33476863 10.1016/j.drugpo.2020.103098 PMC8648280

[R32] Mattson CL, Tanz LJ, Quinn K, Kariisa M, Patel P, Davis NL Trends and geographic patterns in drug and synthetic opioid overdose deaths—United States, 2013–2019. Morbidity and Mortality Weekly Report. 2021;70(6):202. 33571180 10.15585/mmwr.mm7006a4 PMC7877587

[R33] Megerian CE, Bair L, Smith J, Browne EN, Wenger LD, Guzman L, , 2023. Health risks associated with smoking versus injecting fentanyl among people who use drugs in California. Drug Alcohol Depend. 255, 111053. 38128362 10.1016/j.drugalcdep.2023.111053

[R34] National Institute on Drug Abuse. Drug Overdose Death Rates. 2023.

[R35] Ondocsin J, Holm N, Mars SG, Ciccarone D, 2023. The motives and methods of methamphetamine and ’heroin’ co-use in West Virginia. Harm Reduct. J 20 (1), 88. 37438812 10.1186/s12954-023-00816-8 PMC10339587

[R36] Parent S, Papamihali K, Graham B, Buxton JA, 2021. Examining prevalence and correlates of smoking opioids in British Columbia: opioids are more often smoked than injected. Subst. Abus. Treat., Prev., Policy 16 (1), 79. 10.1186/s13011-021-00414-6 PMC8522853 34663374

[R37] Rudolph JE, Cepeda JA, Astemborski J, Kirk GD, Mehta SH, German D, , 2024. Longitudinal patterns of use of stimulants and opioids in the AIDS linked to the Intravenous experience cohort, 2005-2019. Int J. Drug Policy 126, 104364. 38408416 10.1016/j.drugpo.2024.104364 PMC11056308

[R38] Singh SB-G C; Kingston S Distribution of Safer Drug Smoking Supplies as a Public Health Strategy. 2022 January 2022.

[R39] Spencer M, Garnett MF, Miniño AM,. Drug Overdose Deaths in the United States, 2002–2022. Centers for Disease Control and Prevention (CDC) 2024 March 21.

[R40] Sterne JA, 2001. Davey Smith G. Sifting the evidence-what’s wrong with significance tests? Bmj 322 (7280), 226–231. 11159626 10.1136/bmj.322.7280.226 PMC1119478

[R41] Stöver HJ, Schäffer D, 2014. SMOKE IT! Promoting a change of opiate consumption pattern - from injecting to inhaling. Harm Reduct. J 11 (1), 18. 24973031 10.1186/1477-7517-11-18 PMC4094754

[R42] Strathdee SA, Abramovitz D, Harvey-Vera A, Vera CF, Rangel G, Artamonova I, , 2021. Prevalence and correlates of SARS-CoV-2 seropositivity among people who inject drugs in the San Diego-Tijuana border region. PLoS One 16 (11), e0260286. 34807963 10.1371/journal.pone.0260286 PMC8608290

[R43] Syvertsen JL, Ohaga S, Agot K, Dimova M, Guise A, Rhodes T, , 2016. An ethnographic exploration of drug markets in Kisumu, Kenya. Int. J. Drug Policy 30, 82–90. 26838470 10.1016/j.drugpo.2016.01.001 PMC4845648

[R44] Tanz L, Gladden RM, Dinwiddie AT, , 2024. Routes of Drug Use Among Drug Overdose Deaths – United States, 2020-2022. MMWR Morb. Mortal. Wkly Rep 73, 124–130. 38358969 10.15585/mmwr.mm7306a2 PMC10899081

[R45] Valasek CJ, Streuli SA, Pines HA, Strathdee SA, Borquez A, Bourgois P, ,2023. A lotta people switched playing hard ball to playing Russian roulette”: Experiences with rising overdose incidence caused by drug supply changes during the COVID-19 pandemic in the San Diego-Tijuana border metroplex. Drug Alcohol Depend. Rep 7, 100154. 37089868 10.1016/j.dadr.2023.100154 PMC10113744

[R46] Wong CY, Zhu W, Aurigemma GP, Furukawa N, Teshale EH, Huang YA, , 2021. Infective Endocarditis Among Persons Aged 18-64 Years Living with Human Immunodeficiency Virus, Hepatitis C Infection, or Opioid Use Disorder, United States, 2007-2017. Clin. Infect. Dis 72 (10), 1767–1781. 32270861 10.1093/cid/ciaa372

[R47] Zou G, 2004. A modified poisson regression approach to prospective studies with binary data. Am. J. Epidemiol 159 (7), 702–706. 15033648 10.1093/aje/kwh090

